# Active management of the third stage of labor: A brief overview of key issues

**DOI:** 10.4274/tjod.39049

**Published:** 2018-09-03

**Authors:** Kemal Güngördük, Yusuf Olgaç, Varol Gülseren, Mustafa Kocaer

**Affiliations:** 1 Muğla Sıtkı Koçman University, Training and Research Hospital, Clinic of Gynecology and Oncology, Muğla, Turkey; 2 Bilim University Faculty of Medicine, Department of Obstetrics and Gynecology, İstanbul, Turkey; 3 Kaman State Hospital, Clinic of Obstetrics and Gynecology, Kırşehir, Turkey; 4 University of Health Sciences, İzmir Tepecik Training and Research Hospital, Department of Obstetrics and Gynecology, İzmir, Turkey

**Keywords:** Postpartum hemorrhage, active management of the third stage of labor, uterotonic agents

## Abstract

Postpartum hemorrhage is a potentially life-threatening, albeit preventable, condition that persists as a leading cause of maternal death. It occurs mostly during the third stage of labor, and active management of the third stage of labor (AMTSL) can prevent its occurrence. AMTSL is a recommended series of steps, including the provision of uterotonic drugs immediately upon fetal delivery, controlled cord traction, and massage of the uterine fundus, as developed by the World Health Organization. Here, we present current opinion and protocols for AMTSL.

## Introduction and Definitions

Postpartum hemorrhage (PPH) and blood loss complications constitute one of the most common causes of maternal mortality and morbidity. PPH is defined differently in various countries (Table 1)^(1,2,3,4)^. Its incidence is 11% globally among women in labor^(1,2,3,4)^. The third stage of labor (TSL) is defined as the time between the delivery of the baby and the expulsion of the placenta. The duration of the third stage is ~6-30 minute^(3,4)^. The pathophysiology of the TSL is still not fully understood. During this stage, expulsion of placenta with the formation of capillary hemorrhage after the birth of the baby is followed by shrinking of the placental surface with uterine contractions, and finally ends with the discharge of the placenta from the uterus. Hemorrhage is restricted with uterine contractions and activation of the coagulation system^(2,3,4)^. As can be understood from this definition, some degree of hemorrhage always occurs at this stage (~100-250 cc). It is important to limit the amount of hemorrhage to the minimum possible level. Accordingly, the World Health Organization (WHO) suggested the active management of the TSL (Table 2)^(3)^.

### Uterotonic agents

1) Oxytocin: Oxytocin is the most commonly used agent and the primary drug of choice in the TSL. Oxytocin’s action is unique to the smooth muscles of the uterus; it increases the amplitude and frequency of contractions. Oxytocin binds to a G-protein on the surface of uterine myocytes, resulting in the generation of diacylglycerol (DAG) and inositol triphosphate (IP3) via the action of phospholipase C on phosphatidylinositol bisphosphate. DAG stimulates PG synthesis, and IP3 stimulates the release of calcium from the sarcoplasmic reticulum. It also activates cyclooxygenase 2 by a further G-protein interaction and, in doing so, stimulates PG synthesis.

Oxytocin can be used just after delivery of the front shoulder of the baby or expulsion of the placenta. Generally, its administration route and dose are 10 IU intramuscularly (IM). It can also be used intravenously (IV), which is typically preferred during cesarean sections (CS). A newly developed oxytocin tablet has recently been presented that can be applied successfully via the sublingual route^(5)^. An *in vitro* study showed a >30% reduction in tissue transepithelial electrical resistance after treatment with the oxytocin fast-dissolving tablet, implying an increase in the permeability of the mucosal tissue to oxytocin^(5)^. However, it may cause ST depression on an electrocardiogram and hypotension. The efficacy of oxytocin is the same with both administration routes. In a 600-patient study from Turkey, there was no statistically significant difference in the amount of postpartum blood loss between IM and IV administration^(6)^. Oxytocin also decreased postpartum blood loss when applied inside the placental cord^(7)^.

2) Ergometrine (methergine): Ergot alkaloids exert various effects throughout the body on at least three different types of receptor. They are non-selective 5-hydroxytryptamine 1 agonists and have affinities for dopamine and noradrenalin receptors. Ergot alkaloids are absorbed rapidly and completely after oral administration. Both are usually effective within 1-5 min after an IM injection. They are metabolized in the liver, and reported half-lives range from 0.5 to 2 h. Their actions on the uterus are probably a result of their agonist properties against adrenergic a-receptors; these receptors, when stimulated, lead to IP3 release and to calcium mobilization from the sarcoplasmic reticulum. To date, there is only one prospective study in the English literature on this topic. In that study, the authors compared the efficacy of rectal misoprostol 400 µg, oxytocin 10 IU injected IM, methylergometrine 0.2 mg injected IV, and 0.5 mg ergometrine +5 IU oxytocin injected IM in reducing blood loss in the TSL. They found that methylergometrine had the “best” uterotonic drug profile (lowest blood loss during the third stage and duration of the TSL). However, the study had several limitations. Most importantly, it was not a randomized study, the trial was not double-blinded, leading to the possibility of biased results, and no power calculation was reported^(8)^. Ergometrine causes continuous contraction of the uterus. There is not enough evidence about its use as a single agent. It is typically administered at 0.2 mg IM. Its use must be avoided in patients with hypertension.

3) Syntometrine: This contains 5 IU oxytocin and 0.5 mg ergometrine. The time of onset of the uterine response after IM administration is shorter than after ergometrine alone, and the duration of action is several hours. Although it was found to be more effective than oxytocin in a review, the adverse effect profile (hypertension, nausea, vomiting) restricts its use^(9)^.

4) Misoprostol: This is a synthetic prostaglandin E1 derivative. It is an inexpensive drug and is stored readily. It does not cause high blood pressure and can be used in patients with asthma. Its most common adverse effect is flushing. Although the amount of blood loss has been shown to have been reduced with prophylactic use of misoprostol in many studies, it is not as effective as oxytocin. Consequently, oxytocin is the first choice for the prophylaxis of PPH^(10,11,12)^. In countries where the socioeconomic level is very low and home deliveries are common, misoprostol can be used as the first-line drug; it can be used orally, rectally or sublingually. The route of administration and dose differ from country to country. The WHO and International Federation of Gynaecology and Obstetrics recommend a single dose of 600 µg misoprostol, oral or sublingual, for the prophylaxis of PPH^(13)^. According to a recently published meta-analysis result, misoprostol has been used in the third stage of labor to prevent PPH when a sterile syringe and trained midwife were absent^ (14)^. In a prospective randomized trial published in 2016, it was shown that the additional use of buccal misoprostol in conjunction with active management of the TSL reduced the need for additional uterotonic drugs^(15)^.

5) Tranexamic acid: Tranexamic acid (TA) is a lysine derivative with anti-fibrinolytic activity that inhibits fibrin degradation by blocking lysine-binding regions on plasminogen. TA is absorbed from the gastrointestinal tract at 30-40%^(16,17)^. Its plasma half-life is 2 h, and its plasma protein binding ratio is ~3%, which is solely a result of plasminogen binding. It can cross the placenta and be passed to a breastfeeding infant. It is excreted through the urine, so it must be avoided in patients with renal failure. It can be used in an oral, local or parenteral manner. It is typically a well-tolerated drug. Rarely, it may cause nausea, vomiting, hypotension, and dizziness^(17,18)^. These adverse effects are more common when it is given parenterally at high flow rates. One gram of IV TA given within 3 hours of PPH was reported to significantly reduce maternal death and the need for surgery^(19)^. In pill form, it is recommended at a 15-25 mg/kg/dosage every 8 h orally for 5-10 days. There may be gastrointestinal adverse effects. The maximum dose is 3-4 g. It should be given at 10 mg/kg/dosage (maximum 500 mg) with slow infusion every 8 h when given parenterally. Activated prothrombin complex concentrates must be avoided^(17,18)^. Using TA for the treatment of PPH, the incremental cost-effectiveness ratios were found below the lower bound of the cost-effectiveness threshold range^(20)^.

### - Tranexamic acid for the prevention of postpartum hemorrhage after cesarean section

There are 11 randomized controlled trials (RCT) about this topic in the literature^(21,22,23,24,25,26,27,28,29,30,31)^. Except in one study, elective CSs were performed in all patients. In all studies, the amount of blood loss decreased with the use of TA and no adverse effects were reported. The first large study was reported by Gungorduk et al.^ (24)^ from Bakırköy Women and Children’s Hospital in 2011. In total, 660 patients were included in the study and a decrease in the amount of blood loss was seen after the routine administration of 5 IU oxytocin following 20 IU oxytocin in 500 ml RL and TA (1 g IV in 5 min) at the third stage of labor; no adverse effects were reported^(24)^. Another study with 740 patients reported similar results in 2013^(29)^. As can be seen from the results of these studies, administration of TA as an additional agent in the TSL decreased the blood loss.

### - Tranexamic acid for the prevention of prevention of postpartum hemorrhage after vaginal delivery

There are three RCTs on this topic in the literature^(24,32)^. Yang et al.^ (32)^ administered 10 IU oxytocin 10 min after delivery of TA in their study. They reported that TA decreased the incidence of PPH. In a study published in 2013 from Kanuni Sultan Süleyman Training and Research Hospital, İstanbul, Turkey, the TSL was managed actively in all patients. An additional 1 g TA was given to a group of patients in this study. At the end of the study, less blood loss and a lower incidence of PPH was reported in the group using TA^(24)^. Mirghafourvand et al.^ (33)^ reported that administration of 1 g TA after 10 IU oxytocin decreased PPH in a study of 120 pregnant women in 2015. In conclusion, TA decreases the amount of blood loss and the incidence of PPH in patients who are managed actively in the TSL.

### Tranexamic acid adverse effects

In a previous study, the risks of myocardial infarction, cerebrovascular stroke, deep vein thrombosis, and pulmonary embolus were not statistically significantly increased compared with a control group^(16)^. The most common adverse effects are nausea and vomiting. Two patients experienced thromboemboli when receiving high-dose TA in the EXADELI study^(34)^. There is only one reported RCT concerning neonatal adverse effects in the postpartum period. No adverse effects related with TA was reported in this study^(24)^. However, the design of the study was inadequate to assess neonatal adverse effects. It can cross the placenta, so there is a need for large studies with administration before cord clamping.

### Route of administration for uterotonic agents

Although the standard doses of uterotonic agents are given above, the actual dose and administration forms differ in various countries. Table 3 lists four common international guidelines for dose and administration^(29,35)^.

### Delivery of placenta with controlled cord traction

Although this was recommended in the 2007 WHO guidelines, it is described as optional for the active management of the third stage in the 2012 updated guidelines^(13)^. An inexperienced operator may cause serious complications, such as uterine inversion. In WHO studies, it was accepted as ineffective for decreasing blood loss. However, according to a meta-analysis reported in 2015, although the risk of blood loss above 1000 mL was not decreased with controlled cord traction, the mean time of the third stage, the mean blood loss (less than 10 mL), and the risk of blood loss less than 500 mL were all decreased^(36)^. The authors noted that controlled cord traction still had a place in active management when performed by experienced personnel. It is also a recommended method for CSs^(24,37)^.

### Uterine fundal massage after placental expulsion

Uterine fundal massage after placental expulsion provides uterine contractions by stimulating endogenous prostaglandin secretion. This method was recommended in the 2007 WHO guidelines, and was described as optional for the active management of the third stage in the 2012 updated guidelines^(13)^. Similarly, Chen et al.^ (38)^ published results of a study of 2340 pregnancies, which showed that the addition of fundal massage to oxytocin did not decrease PPH.

### Possible adverse effects of active management

Adverse effects related to uterotonic agents

- Hypertension, nausea, vomiting due to ergot alkaloids

Risk of placental retention

Neonatal risks related to early cord clamping

- Iron-deficiency anemia

- Intraventricular hemorrhage

- Hypotension

## Results

According to the most recent Cochrane analysis, active management of the TSL decreases the risk of postpartum bleeding of over 1000 mL^(39)^. The possible risks and benefits of active management must be explained to pregnant women and informed consent must be obtained. TA administration, as an extra drug for pregnant women whose TSL is being managed actively, decreases both the amount of blood loss and the incidence of PPH.

## Figures and Tables

**Table 1:**
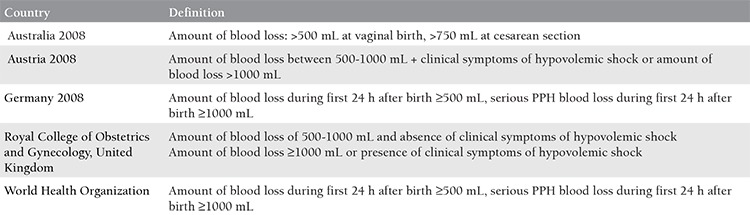
Post-partum hemorrhage definitions

**Table 2:**
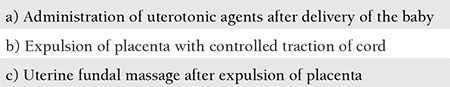
Active management of the third stage of labor

**Table 3:**
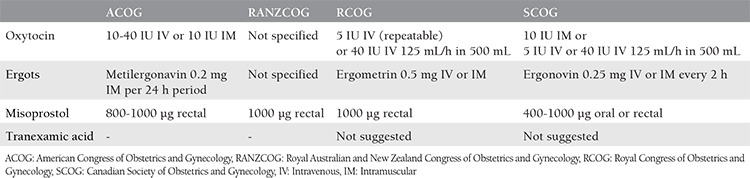
Drug doses and administration forms in four major international guidelines
